# Artificial intelligence and job performance of healthcare providers in China

**DOI:** 10.3389/fpubh.2024.1398330

**Published:** 2024-08-08

**Authors:** Qi Zheng, Yun Jin, Xinying Xu

**Affiliations:** ^1^School of Labor Economics, Capital University of Economics and Business, Beijing, China; ^2^Department of Hepatobiliary Surgery, The First People’s Hospital of Yunnan Province, Kunming, China; ^3^The Affiliated Hospital of Kunming University of Science and Technology, Kunming, China; ^4^School of Insurance and Economics, University of International Business and Economics, Beijing, China

**Keywords:** artificial intelligence, job performance, healthcare providers, patients’ support, China

## Abstract

**Introduction:**

This study explores the influence of artificial intelligence (A.I.) applications on the job performance of healthcare providers, based on data from standardised-trained residents in the First People’s Hospital of Yunnan Province in China.

**Methods:**

The ordinary least squares model is employed to examine the relationship between A.I. applications and job performance. To address potential endogeneity and missing variables, we utilise the propensity score matching method and alternative regression models.

**Results:**

The findings indicate that the job performance of standardised-trained residents positively correlates with A.I. applications. This relationship remains robust after addressing endogenous and missing variables. Further discussion reveals that patients’ support mediates the relationship between A.I. and job performance. Under identical conditions, the job performance of female residents empowered by A.I. is found to be significantly better than that of their male counterparts. Conversely, no heterogeneity is observed regarding the impact of A.I. on the job performance of medical practitioners and clinical medical technicians.

**Discussion:**

This study underscores the positive role of A.I. applications in enhancing the job performance of standardised-trained residents. The results highlight the mediating role of patient support and suggest gender-based differences in the efficacy of A.I. empowerment.

## 1 Introduction

There has been a chronic shortage of healthcare providers internationally (1), primarily driven by a series of factors concerning long-term under-investment in the education of healthcare workers, insufficient training, recruitment problems, and labour market constraints. This shortage, therefore, has increased the workload and related burnout of the healthcare staff, especially during the COVID-19 pandemic (2). This, in turn, tends to enhance the turnover rate, leads to the growing graduates’ reluctance to choose to work as healthcare providers, and dramatically reduces the efficiency and effectiveness of healthcare (3). In this context, attention worldwide has been paid to integrating healthcare work with emerging technologies, among which artificial intelligence (A.I.) plays an essential role in effective and efficient healthcare provision (4).

A.I., considered a part of our daily life, has moved from science fiction to the truth during the last decade, influencing the healthcare system considerably. There has been controversy about the meaning of A.I. The concept of A.I. was first proposed at the Dartmouth Conference in 1956. Its recent connotation has been derived into a series of computer programmes that can simulate, expand, and extend human intelligence (5). The relatively authoritative definition in China comes from the *Standard White Book of Artificial Intelligence* (6), which holds that A.I. is a kind of theory, method, technology, and application system to simulate, extend, and expand human intelligence to perceive the environment, acquire knowledge, and use knowledge to obtain the best result, via digital computers or machines controlled by digital computers. Healthcare A.I. refers to the application of A.I. theory and technology in healthcare settings.

From automated administrative tasks to automated imaging, AI-powered surgical robots, clinical decision aids, and intelligent medicine design, various A.I. tools aiming at mimicking the cognitive functions of human beings (7) have been widely deployed in the healthcare system. Not only in the developed economies but also in China, A.I. application—decreasing cost as well as making great improvement in healthcare outcomes—has obtained interest and momentum. In April 2018, the General Office of China’s State Council (8) issued the *Opinions on Promoting the Development of “Internet* + *Medical and Health”*; moreover, in 2021, the National Development and Reform Commission of China (9) issued the *Implementation Plan for the Construction of High-quality and Efficient Medical and Health Service System during the 14^th^ Five-Year Plan*, which vastly boosted A.I. applications in China’s healthcare systems, where A.I. has been adopted in multiple areas, concerning mining health records, administration assistance, treatment plan designation, playing the role of consultant, and so on.

Per popular belief, A.I. applications will be able to promote early disease detection, facilitate disease surveillance, create new treatments, and make improvements in diagnosis (10). However, there is profound fear that A.I. may cause certain healthcare jobs to become redundant, which will disrupt the provider–patient relationship (11). Additionally, some predictions say that, by 2053, A.I. tools may outperform human healthcare providers during surgery (12), leading to the underperformance of healthcare providers (11). Yet, some others claim that the application of A.I. can reduce repetitive jobs and tasks to improve healthcare providers’ job performance efficiently (taking care of a larger number of patients) and effectively (providing higher quality medical service for patients) (3), clearing the way for human-to-human bonding and emotional intelligence application (10). Therefore, some questions arise. What impact will A.I. applications have on healthcare providers’ job performance? How do A.I. applications affect the performance of providers? Is there heterogeneity in the effect of providers based on gender and professional type difference? This article will discuss these questions.

Due to the massive data in terms of clinical pathological images, Internet of Things tools, and continuous biometric data available for facilitating the deep learning algorithms, research about A.I. applications in the healthcare area has increased substantially (10). Nevertheless, previous studies on A.I. applications in healthcare settings are primarily qualitative, focusing on the topic of macro-structural changes (13–15), management scheme transformation (16), and general pros and cons brought about by A.I. applications, where the large part of them pay attention to the issue that human providers may be replaced by AI-powered technologies (13, 17, 18). However, the main research on the effects of A.I. on performance has concentrated on the employees within enterprises, mainly in the context of manufacturing or business, but rarely on the healthcare providers in hospitals. Few scholars conducted an in-depth quantitative study about the influence of A.I. applications on healthcare providers’ job performance in hospitals, which is probably the marginal contribution of this study. In practice, we attempt to prove that the use of artificial intelligence has a positive impact on improving healthcare providers’ job performance and optimising patients’ treatment experience and that it is conducive to alleviating the dilemma of insufficient medical resources at the macro level.

## 2 Literature review

### 2.1 Concept of job performance

Employee job performance is the core factor driving enterprises to achieve long-term competitive advantages, thus attracting great attention in the academic world. As for the concept of employee job performance, Campbell et al. (19) propose a precise definition advocating that job performance is “the goal-relevant actions of an employee,” meaning whether employees’ behaviour matches organisational goals and whether organisations’ desired results can be achieved by their employees. However, there is no consensus on the definition of employee job performance. Some scholars define it as an employee’s expertise in performing duties to help their organisation achieve its aims and goals (20–22), while some others refer to it as an individual’s productivity in comparison with their colleagues on a set of directly or indirectly work- or task-related behaviours and outcomes (23, 24). Additionally, some researchers indicate that job performance can be seen as the effectiveness of how an individual employee uses the influence opportunity (25), which implies how effectively employees perform their tasks for organisations. To sum up, job performance, in this study, can be defined as the efficiency and effectiveness by which an individual employee conducts their responsibilities for their organisations to achieve organisational goals. From the view of the outcome, job performance is the primary representation of work output, which can be reflected by the quality and quantity of work completed as part of assigned responsibilities (26).

### 2.2 A.I. applications and healthcare providers’ job performance

With A.I. obtaining momentum, enthusiasm for its prospects has been growing. Yet, there have been excessive concerns about healthcare jobs being redundant due to A.I. applications.

Regardless of the uncertainty of A.I. adoption, many scholars insist that there is considerable potential for A.I. to augment the job performance of healthcare providers, both in efficiency and effectiveness. Some scholars indicate that A.I. may be able to complete repetitive and time-consuming tasks or at least make working processes more efficient so that healthcare providers can get more time to focus on their patients’ needs (27). Moreover, healthcare providers accompanied by A.I. can manage care for more patients than those without it, which is termed as “augmented intelligence” (28). It is reported that the productivity of nursing has enhanced by 30–50% due to the adoption of A.I. tools (29). High workload has been seen as an irremovable tag by healthcare providers. Heavy workload brings about substantial time pressure to the healthcare staff (30), while A.I. can release them from huge burden through open-ended clinician notes, data querying from previous records, and transcription of records on patients’ experiences. Additionally, AI-enabled tools, such as deep learning techniques and medical imaging, can facilitate diagnosis decisions, improve therapeutic outcomes, and cut down the diagnostic errors (31). A.I. is useful for processing large amounts of data, which is hard for healthcare providers to handle perfectly, develop new treatment methods, and monitor patient outcomes. Health service quality can thus be improved, increasing patient satisfaction and follow-up rate (7). A.I. can make it more convenient for patients with chronic diseases to obtain health-related information and establish contact with healthcare staff (3). For instance, AI-powered home health monitoring tools enable providers to extend their healthcare services beyond office hours and outside hospitals while facilitating patients’ self-management (3). Furthermore, the complexity of current medical and diagnostic processes requires healthcare providers to work in a collaborative manner that requires strong and effective communication channels through which decision-sharing, action coordination, and outcome evaluation can be conducted (32). A.I. can integrate large amounts of structured and unstructured data from various sources, promoting collaboration among healthcare providers.

However, other scholars indicate that A.I. has generated new problems and challenges to healthcare providers, which may hamper job performance. First, the fear of being overtaken by A.I. tools can negatively impact providers’ job performance, reducing their work efficiency and effectiveness (11, 12), probably due to their low perception of the ability to perform tasks, while self-compared with A.I.-enabled technologies (2). According to Wang et al. (33), the application of A.I. technology changes the organisation’s external environment, which significantly impacts employees’ survival and development, bringing them job insecurity. Facing the complexity of emerging technology, employees may show negative emotions, while job insecurity will harm their job performance. Second, it is possible for healthcare providers to over-rely on A.I. tools, and, therefore, too complacent to make errors in the healthcare process (34). Third, A.I. may be able to improve work efficiency and quality but lacks human traits, such as empathy, compassion, and other emotions, which may reduce patient satisfaction and follow-up rate, thus affecting the healthy development of the provider–patient relationship (35).

In general, there are two views on A.I. impact on the job performance of healthcare providers. The supportive view believes that applying A.I. can promote providers’ job performance, which we strongly support. The opposite view is that using A.I. will bring fear and anxiety, reducing individual job performance and ruining the provider–patient relationship. However, the existing research on this topic, in the medical setting, are limited to the qualitative study, the literature review, and the theoretical discussion, without providing solid empirical evidence from quantitative approaches.

As far as our concern, job performance measures the degree to which employees’ behaviours and outcomes are close to work or organisational goals, which is positive feedback provided to employees. According to the self-determination theory, individual self-management and self-decision-making tendencies will guide employees to engage in activities that benefit their job performance improvement (36). In practice, resources are unevenly distributed and are very limited, while the roles and needs of employees are diversified. To maximise the value of resources, based on the resource conservation theory, employees may be more willing to invest resources in behaviours with low risk but high return rate (37); thus, learning with convenience, accessibility, low risk, and the high return rate is considered the ideal behaviour. After employees realise that A.I. applications may replace those individuals without innovative knowledge and skills to meet the demand of their current job, they are bound to increase their resource investment in learning how to use the new technology (13, 38), which will eventually improve their job performance. Specifically, when technological changes lead to changes in the working environment and conditions, the requirements for the working skills of employees arise when they are urged to integrate internal and external resources which can be invested to improve their skills and strengthen their professional knowledge (13, 38). The improvement of skill level and the accumulation of knowledge can promote the advancement of individual job performance (39). In addition, Hazarika (3) believes that the rapid processing of procedural work by A.I. can free employees from tedious and repetitive work and enable them to focus more on core tasks, thus improving overall job performance.

Therefore, this study supposes that A.I. application positively affects healthcare providers’ job performance. Moreover, to some extent, the patients’ support is an essential external resource for healthcare providers’ work. Hence, the question of “whether most of your patients support you to utilise A.I. during the treatment?” may influence the mechanism between A.I. application and healthcare providers’ job performance; that is A.I. can impact providers’ job performance through support from their patients.

Moreover, women are more engaged than men in non-routine tasks favouring cognitive skills, such as empathy and social skill, but less involved than men in routine tasks, which can be completed quickly by A.I (40). Therefore, with the help of A.I. technology, female healthcare providers’ performance in both routine and non-routine tasks improves significantly, while the positive effect of A.I. use on male healthcare providers’ performance may be less than that of their female counterparts (41–44). Further, given the differences in the capability demand of different professions, there might be heterogeneity in the impact of A.I. on job performance among different categories of healthcare providers (45, 46).

To sum up, this study predominantly discusses how the application of A.I. impacts healthcare providers’ job performance, the mechanism of this effect, whether patients’ support plays a mediating role, and the impact of heterogeneity by gender and professional type.

## 3 Data and methods

### 3.1 Data

The data utilised comes from a study survey conducted by the Capital University of Economics and Business and the First People’s Hospital of Yunnan Province between August 5 to August 30, 2022. This coincides with the post-pandemic period, wherein the workload of hospitals has increased significantly. In this context, hospitals have increased their investment and applications related to A.I. Therefore, this study seeks to build a clearer picture of the utility of A.I. The survey explored in depth the influence of A.I. applications on the job performance of standardised-trained residents in the hospital. Online questionnaires were sent via Wenjuanxing (an online questionnaire distribution platform: https://www.wjx.cn/) to two main types of standardised-trained residents—medical practitioners and clinical medical technicians—in the First People’s Hospital of Yunnan Province. The questionnaires covered information about residents’ individual and family characteristics, human capital characteristics, A.I. applications, job performance, working conditions, and patients. A sample of 417 participants were recruited. After excluding participants that did not meet the study inclusion criteria or with extreme and missing values, 344 valid responses remained; these 53 (417–344) participants had actually participated in the survey, but were excluded to ensure the reliability of the research results, partly because of omissions or extreme values provided by them.

Based on previous research and our definition, job performance, the *dependent variable* in the analysis, is measured from two dimensions: the quality and quantity of tasks completed as part of the assigned responsibilities (26), representing the effectiveness (25) and efficiency (23, 24) of standardised-trained residents’ hospital duties. Specifically, work efficiency is a continuous variable, measured as the number of patients treated per day, with the statistics showing that there is an average of about 8.195 patients treated per day for every resident; work effectiveness is measured from the patient’s perspective, including the patient satisfaction and follow-up rate (%). Patient satisfaction was assessed using the question, “Have your patients praised you for an outstanding performance last month?” and was denoted as 1 when they answer “have been praised by patients last month” (denoting patients’ satisfaction with residents) and 0 when they “have not been praised by patients last month” (denoting patients’ dissatisfaction with residents). A total of 132 residents (38.37%) reported having been appraised, while 212 (61.63%) stated otherwise. The follow-up rate of patients is a continuous variable measured as a percentage, with a mean rate of 42.3% (maximum 100%). In addition, based on the two aforementioned dimensions above, we also calculated the comprehensive index of job performance (a continuous variable) through principal component analysis (PCA), which is a statistical method wherein a group of possibly correlated variables is transformed into a group of linearly uncorrelated variables by orthogonal transformation, which is called the principal component.

A.I. application, the *independent variable*, was measured by the question, “Have you applied A.I. tools to your work (such as diagnosis)?” and is denoted by 1 when the answer is “have” and 0 when it is “have not.” As reported, 45.06% of residents have applied A.I. technologies, whereas 54.94% have still not used them.

The *influencing mechanism (mediator)* is the patients’ support for A.I. application, as the external resources (13, 38) can be measured by “Do most of your patients support you to utilise A.I. during the treatment?” transformed into a dummy variable. The answer “yes” takes the value of 1, whereas the response “no” takes the value of 0. Statistics indicate that 185 residents have obtained their patients’ support for A.I. applications, which is more than half the total. However, 159 reported they had not received patient support on A.I. usage (46.22%).

**Figure 1:**
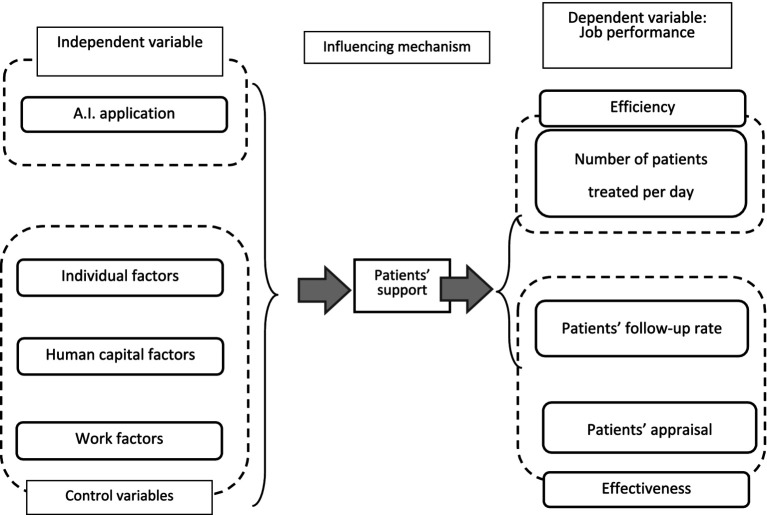
Conceptual framework.

*Control variables* include individual factors (age, gender, and Hukou status [“Hukou” signifies a worker’s residency registration status in China, which imposes restrictions on migrant workers with regard to searching for and obtaining employment]), human capital factors (education levels and work experience), and work factors (professional categories: medical practitioners or clinical medical technicians). The conceptual framework is shown in Figure 1.

Table 1 presents the definition and statistical description of the variables involved in this study, reporting the mean and standard deviation of continuous variables and the percentages of categorical variables.

**Table 1:** Variable definition and description.

Variables			Definitions	Frequency	Proportion	Min	Max
				(Mean)	(S.D.)		
The independent variable	The A.I. application	Whether the A.I. is applied for work	Have you applied A.I. tools to your work?				
			Yes = 1	155	45.06%	——	——
			No = 0	189	54.94%	——	——
The dependent variable	Job performance	Work efficiency (quantity)	Daily number of patients treated	8.195	11.33	0	50
		Work effectiveness (quality)	Patient’s appraisal: Have been praised by patients for outstanding performance last month?				
			Yes = 1	132	38.37%	——	——
			No = 0	212	61.63%	——	——
			Follow-up rate of patients	0.423	0.34	0	1
Control variables	Individual factors	Age	2022 - year of birth (year)	26.11	2.884	22	56
		Gender	Male = 1	154	44.77%	——	——
			Female = 0	190	55.23%	——	——
		Hukou status	Local = 1	149	43.31%	——	——
			Non-local = 0	195	56.69%	——	——
	Human capital factor	Education level	Years for education	15.68	1.604	14	22
		Working experience	Months of working at the hospital	15.08	8.888	1	60
	Work factor	Categories of standardised-trained residents	Medical practitioners = 1	246	71.51%	——	——
			Clinical medical technicians = 0	98	28.49%	——	——
Influencing mechanism		Patients’ support	Have = 1	185	53.78%	——	——
			Have no = 0	159	46.22%	——	——
The variable for robust test	Alternative independent variable	A.I. application frequency	Frequency of A.I. tools applied per week	3.548	4.421	0	24

### 3.2 Methods

#### 3.2.1 Ordinary least squares model

Ordinary least squares regression (OLS) is a common technique for estimating coefficients of linear regression equations that describe the relationship between one or more independent quantitative variables and a dependent variable (simple or multiple linear regression). Least squares stand for the minimum squares error (SSE). Maximum likelihood and generalised method of moments estimators are alternative approaches to OLS. OLS can be defined as a linear regression technique used to estimate a model’s unknown parameters. The method relies on minimising the sum of squared residuals between the actual (observed values of the dependent variable) and predicted values from the model.

Job performance is the outcome of interest for the study, where the comprehensive index of job performance is a continuous variable. Thus, we employ the OLS model to explore the effect of A.I. application on the job performance of standardised-trained residents (7). The performance determination model is as follows:


(1) $$\mathrm{JobPerfor}m_{i}=\beta_{0}+\beta_{1}AI_{i}+\beta_{2}\mathrm{xi}+\sum \beta_{i}x^{'}+\mu_{i}\left(i=0,\ldots n\right)$$

where $\mathrm{JobPerfor}m_{i}$ is the dependent variable, measured by the comprehensive index of job performance; $AI_{i}$ is the independent variable denoting applying A.I. tools; $x_{i}$ represents a vector of a series of control variables; and $\beta_{0}$ is the intercept term, $\beta_{1}$ the coefficient vector of $AI_{i}$ , $\beta_{2}$ the coefficient vector of other control variables, $\sum \beta_{i}x^{'}$ the other possible omitted variables, and *μ*_i_ the random error term.

#### 3.2.2 Mediation (influencing mechanism) analysis

It is speculated that the effect of A.I. application may be mediated by patients’ support, finally influencing individuals’ job performance (13, 38). Therefore, patients’ support can be the influencing mechanism of the impact of A.I. on residents’ job performance. To further explore the mechanism, this analysis introduces patients’ support for A.I. application as the influencing mechanism between the independent and dependent variables.

Based on the traditional approach of testing mediating variables proposed by Baron and Kenny (47), the regression equations are established as follows:


(2) $$\mathrm{JobPerfor}m_{i}=\beta_{0}+\beta_{1}AI_{i}+\beta_{2}x_{i}+\sum \beta_{i}x^{'}+\mu_{i}\left(i=0,\ldots n\right)$$

(3) $$\mathrm{Patien}t_{i}=\alpha_{0}+\alpha_{1}AI_{i}+\alpha_{2}z_{i}+\sum \alpha_{i}z^{'}+\varepsilon_{i}$$

(4) $$\mathrm{JobPerfor}m_{i}=\chi_{0}+\beta_{1^{\prime }}AI_{i}+\chi_{1}\;\mathrm{Patien}t_{i}+\chi_{2}z_{i}+\sum \chi iz^{'}+\delta_{i}$$

where $AI_{i}$ is the mediating variable, and z is the control variable. The coefficient $\beta_{1}$ of Equation (2) is the total effect of A.I. application on residents’ job performance; the coefficient of $\alpha_{1}$ Equation (3) is the effect of A.I. application on the mediator (influencing mechanism)—patients’ support; the coefficient $\chi_{1}$ of Equation (4) is the effect of the mediator (influencing mechanism) on residents’ job performance after controlling for other variables, and the coefficient $\beta_{1^{\prime }}$ is the effect of A.I. application on performance after controlling for the mediator. The mediation effect is usually tested by three methods, namely the stepwise method, the Sobel test, and the bootstrap method. This study adopted the stepwise approach, followed by the bootstrapping procedure to test the significance of mediation effects.

## 4 Results

### 4.1 Impact of A.I. application on job performance

The OLS regression was performed first to quantify the impact of A.I. application on standardised-trained residents’ job performance. Table 2 displays the results of OLS regression for a comprehensive index of residents’ job performance, with Model 1 controlling for individual factors, Model 2 controlling for individual and human capital factors, and Model 3 considering all control factors.

**Table 2:** Results for OLS regression of comprehensive index of job performance.

Job performance	Model 1	Model 2	Model 3
A.I. application (Yes = 1; No = 0)	0.2212***	0.2269***	0.2278***
	(0.0668)	(0.0661)	(0.0663)
Age	0.0098	0.0043	0.0042
	(0.0115)	(0.0118)	(0.0118)
Gender (Male = 1; Female = 0)	0.0558	0.0340	0.0343
	(0.0669)	(0.0663)	(0.0664)
Hukou status (Local = 1; Non-local = 0)	0.0506	0.0263	0.0268
	(0.0667)	(0.0664)	(0.0665)
Education level		0.0525**	0.0518**
		(0.0207)	(0.0209)
Working experience		0.0076*	0.0075*
		(0.0038)	(0.0039)
Categories of standardised-trained residents (Medical practitioners = 1; Clinical medical technicians = 0)			0.0213
			(0.0729)
Constant term	−0.4004	−1.1784***	−1.1796***
	(0.3027)	(0.4470)	(0.4476)
Observations	344	344	344
*Adj R^2^*	0.2262	0.2523	0.2897

*, **, *** represent significance at the 10, 5, and 1% levels, respectively. The figures in brackets are standard errors.

Models 1, 2, and 3 regression results show that the influence of A.I. applications on the comprehensive index of job performance is positively significant at the 1% level, with coefficients of 0.2212, 0.2269, and 0.2278, respectively. Results from Model 3 indicate that, compared with those without help from A.I., residents who adopt A.I. tools can significantly enhance healthcare providers’ job performance by 22.78%, which is in line with the evidence from previous research (3, 7, 27–30, 32).

Table 3 presents the results of the OLS regression and the probit regression for the effect of A.I. application on residents’ job performance considering the dimensions of efficiency and effectiveness. Model 4 is the OLS regression model for the daily number of patients treated, Model 5 is the OLS regression model for follow-up rate, and Model 6 is the probit regression model for patients’ appraisal.

**Table 3:** Results from OLS and probit regressions of job performance, respectively, for two dimensions.

Job Performance	Model 4	Model 5	Model 6
	Daily number of patients treated (OLS)	Follow-up rate (OLS)	Patient’s appraisal (Probit)
A.I. application	2.6840**	0.0824**	0.3961***
	(1.2180)	(0.0366)	(0.1421)
Individual variables	Controlled	Controlled	Controlled
Human capital variables	Controlled	Controlled	Controlled
Work variables	Controlled	Controlled	Controlled
Constant term	−11.5047	0.1385	−1.2092
	(8.2272)	(0.2469)	(0.9513)
Observations	344	344	344

*, **, *** represent significance at the 10, 5, and 1% levels, respectively. The figures in brackets are standard errors. OLS, ordinary least squares.

Results from Models 4, 5, and 6 demonstrate that A.I. application has a positive correlation with the daily number of patients treated, the follow-up rate as well as the patients’ appraisal, with coefficients of 2.6840, 0.0824, and 0.3961, respectively, at the significance levels of 5, 5, and 1%. It is implied that applying A.I. can benefit residents in terms of nearly 2.7% more patients treated per day, an increase of 8.24% in the monthly follow-up rate, and 39.61% more possibility of being appraised by patients. Model 6 has an *Adj R^2^* value of 28.97%, standing for it explains 28.97% of the data variability.

The results as mentioned above are consistent with those in existing studies, with opinions that A.I. can: release residents from repetitive, time-consuming, and heavy work to pay more attention to their patient’s needs (27, 30); extend residents’ provision of healthcare services beyond office hours and outside hospitals; help patients’ self-management (3); improve therapeutic outcomes and reduce diagnostic errors (31); promote convenience for patients with chronic diseases to get health-related information and build up contact with residents (3); and help residents work in a more collaborative manner (32), therefore driving work efficiency, keeping good relations, and providing more high-quality healthcare treatment to their patients. Based on the self-determination theory (36) and resource conservation theory (37), when technological changes lead to changes in the working environment and conditions, the healthcare skills and knowledge requirements increase (13, 38), which motivates residents to keep learning, with low risk and high return. The advancement of skill and knowledge will eventually promote residents’ job performance (39).

### 4.2 Robustness test

To test whether the above conclusions are robust, we conduct the robustness test using the following methods: First, the propensity score matching (PSM) approach is adopted to solve the problems of self-selection and missing values; Second, the probit model is used as an alternative method for OLS to perform the regression analysis; Third, we also change the measurements of independent variables to verify our results.

**Figure 2:**
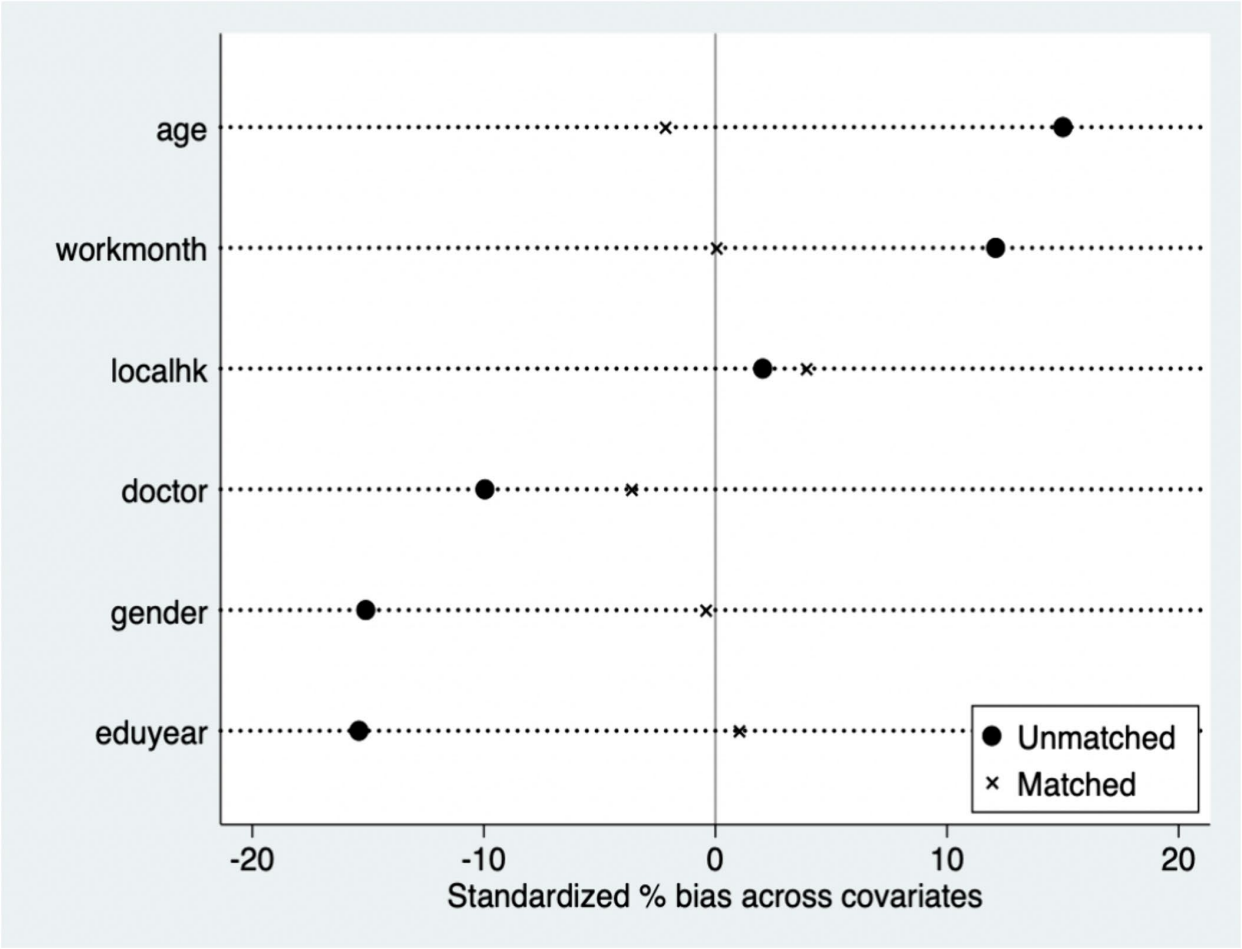
Balancing test for radius matching.

**Figure 3:**
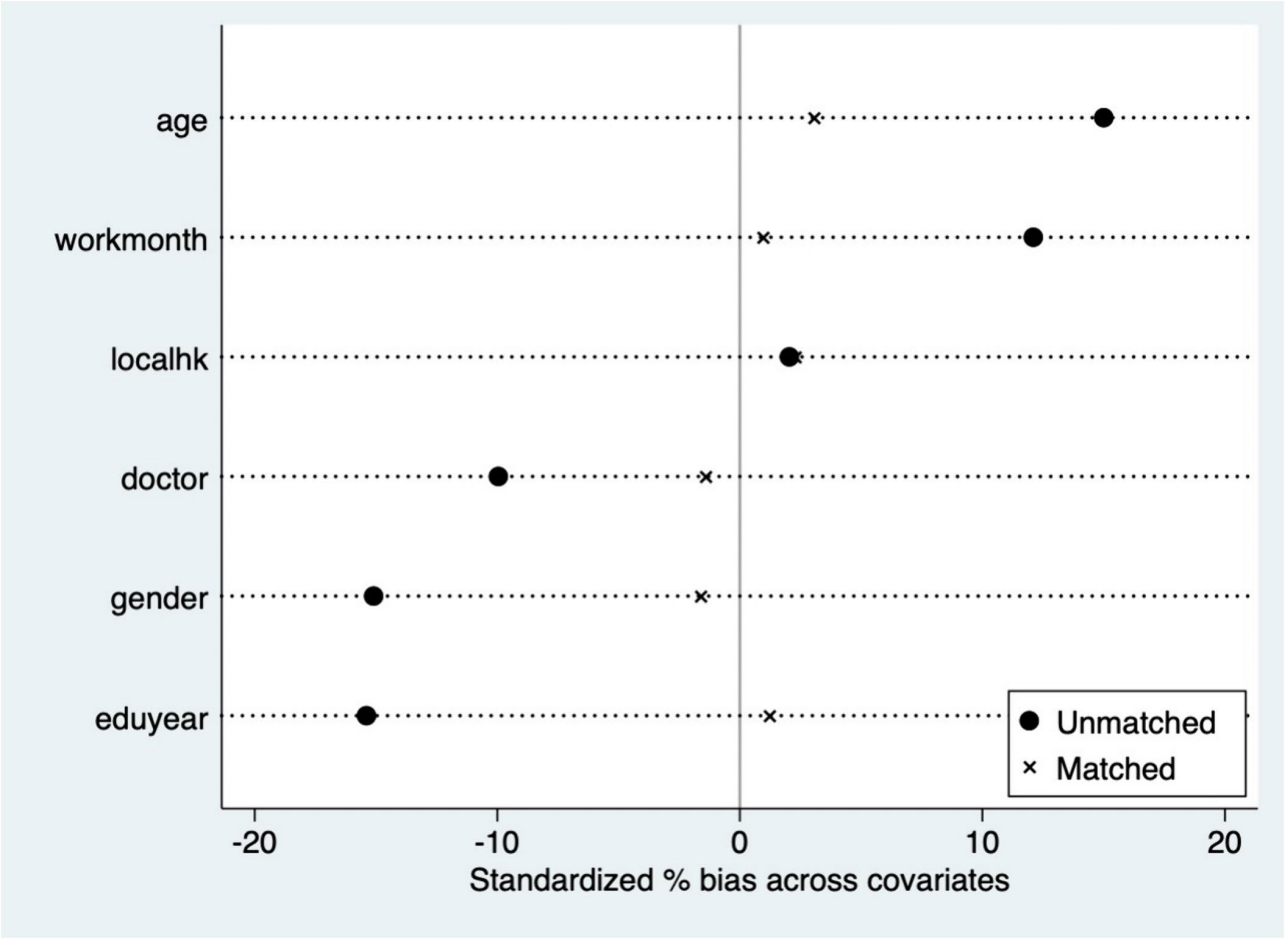
Balancing test for kernel matching.

The PSM method is used for further robustness tests. The research initially performs a balancing test, discovering a substantial difference between groups with varying reproductive circumstances, with inconsistencies in each factor. Taking radius matching and kernel matching as examples (Figures 2, 3), suggesting that after the propensity scores are matched, the inconsistencies of these variables except localhk are significantly reduced, and the sample averages are significantly closer than before, suggesting that the balancing test was passed. We analyse the average treatment effect for the treated (ATT) for the effect of A.I. applications on residents’ job performance via PSM. Table 4 shows the average treatment effect (ATT) of parenthood status on the hourly platform income of gig workers. Given the bias in the standard error of single matching, the self-sampling bootstrap method is used to modify the standard error in this case. Subsequent to controlling for control and treatment group sample bias, the results demonstrate that the average treatment effect achieved by various matching approaches is substantial.After considering self-selection, the effect of A.I. applications on job performance remains significant, and the average treatment effect obtained by nearest neighbour matching (1:1), nearest neighbour matching (1:4), kernel matching, and radius matching is close, confirming the robustness of the above-mentioned findings.

**Table 4:** Average treatment effect (ATT) results under different matching methods.

Method	ATT	Standard Error	*t* value
Nearest Neighbor Matching(1:1)	0.326***	0.1012	3.22
Nearest Neighbor Matching(1:4)	0.2262***	0.0827	2.74
Radius Matching	0.2297***	0.0747	3.07
Kernel Matching	0.2185***	0.0707	3.09

*, **, *** represent significance at the 10, 5, and 1% levels, respectively. The figures in brackets are standard errors.

In addition, we change the measurement of the independent variable from A.I. application to the frequency of A.I. application, that is, ‘frequency of A.I. tools applied per week’, and replace the OLS regression with probit regression to further validate the robustness of the findings. The study makes the regression for frequency of A.I. application and the probit regression, results of which are mostly consistent with our prior results, reaffirming their robustness.

## 5 Further discussion: influencing mechanism and heterogeneity analysis

### 5.1 Mediation (influencing mechanism)

A.I. applications might impact residents’ job performance via their patients’ support (13, 38), which is regarded as an essential external resource for performance enhancement and is the influencing mechanism of the relations between A.I. and job performance. Table 5 reports the test results from the effects of A.I. applications on job performance, mediated by patients’ support and the test results for the effect of the influencing mechanism by bootstrapping method.

**Table 5:** Influencing mechanism (mediation) analysis: Patients’ support.

Variables	Model 7	Model 8	Model 9
	Job performance	Patients’ support	Job performance
A.I. application	0.2278***	0.4076***	0.1534**
	(0.0663)	(0.0502)	(0.0719)
Individual variables	Controlled	Controlled	Controlled
Human capital variables	Controlled	Controlled	Controlled
Work variables	Controlled	Controlled	Controlled
Patients’ support			0.1827**
			−0.0714
Constant term	−1.1796***	0.4921	−1.2695***
	(0.4476)	(0.3392)	(0.4453)
Observations	344	344	344
Results of bootstrapping (patients’ support)			
Mediation effect coefficient (bs_1)	0.0744^**^(Z = 2.39)		
Direct effect coefficient (bs_2)	0.1534^**^(Z = 2.12)		
The proportion of the mediation effect	0.3268		

*, **, *** represent significance at the 10, 5, and 1% levels, respectively. The figures in brackets are standard errors.

Based on the basic regression, the first step in the influencing mechanism test is to conduct the regression of the effect of A.I. applications on patients’ support using the binary probit model (Model 8). Subsequently, we include patients’ support in the job performance regression (Model 9) using the OLS regression to observe how the coefficients of residents’ job performance change.

Model 8 demonstrates that A.I. applications significantly and positively affect patients’ support at the 1% significance level. As per the results from Model 9, the coefficient of patients’ support is 0.1827, revealing significant impacts on job performance at the 5% significance level. Moreover, A.I. application is still positively related to job performance at the 5% level. However, there seems to be a decrease in its coefficient, from 0.2278 to 0.1534, which proves that the patients’ support mediates the influence of A.I. application on the job performance of standardised-trained residents in the hospital. Testing via bootstrapping method shows that the mediation effect is significant at the 5% level.

### 5.2 Heterogeneity analysis by gender and professional type

We argue that the gender (41–44) and the professional type (medical practitioners and clinical medical technicians) (45, 46) of healthcare providers also differ in the impact of A.I. applications on their job performance. This section mainly analyses whether the effect of A.I. application on residents’ job performance is heterogeneous between men and women and between medical practitioners and clinical medical technicians. To explore further, we constructed the interaction items of *A.I. application × Male* and ***A.I. application × Medical practitioners***, aiming to focus on whether the interaction items significantly affect job performance. Table 6 presents the regression results for the heterogeneity analysis.

**Table 6:** Heterogeneity analysis of gender and categories of standardised-trained residents.

Job Performance	Model 10	Model 11
	Male (Female = 0)	Medical practitioners (Clinical medical technicians = 0)
A.I. application	0.3689***	0.1617
	(0.0879)	(0.1232)
Individual variables	Controlled	Controlled
Human capital variables	Controlled	Controlled
Work variables	Controlled	Controlled
A.I. application×Male	−0.3193**	
	(0.1317)	
A.I. application × Medical practitioners		0.0934
		(0.1466)
Constant term	−1.2367***	−1.1617**
	(0.4450)	(0.4489)
Observations	344	344

*, **, *** represent significance at the 10, 5, and 1% levels, respectively. The figures in brackets are standard errors.

Results from Models 11 and 12 indicate that, when applying A.I. tools for work, female residents may perform 31.93% better than their male counterparts, implying that AI-enabled tools may bring more performance improvement for female healthcare providers, in line with previous research (41–44). Female healthcare providers’ abilities to do repetitive tasks may be weaker than that of their male counterparts in the work process (40); these tasks can be completed by A.I. technologies efficiently and effectively. However, the advantage of women over men is that they are more sensitive and have better people skills and a stronger drive to empathise, which are difficult to be replaced by technological advances and are even complementary to technological change (41–44). Therefore, when female healthcare providers are empowered by A.I., their level of performance improvement may be much higher than that of their male counterparts. However, there seems to be no significant difference between A.I. impacts on the job performance of medical practitioners and clinical medical technicians, probably because there may only be a slight difference in the routine and non-routine tasks and the cognitive and non-cognitive abilities between both categories of residents.

## 6 Conclusion

Existing research has yielded a great deal of data on the characteristics and trends of A.I. applications in healthcare, mainly qualitative studies, focusing on the macro-structural and management scheme changes, as well as the pros and cons of applying A.I.-enabled technologies. This study has made a marginal contribution to the previous research as it is an in-depth empirical quantitative analysis of A.I. application’s influences on healthcare providers’ job performance. Furthermore, the influencing mechanism and the impact heterogeneity by gender and professional categories have also been analysed.

Based on the analysis of data from standardised-trained residents in the First People’s Hospital of Yunnan Province in China, this study draws some conclusions. Primarily, there seems to be a positive relationship between A.I. application and the standardised-trained residents’ job performance in terms of the job performance comprehensive index, number of patients treated per day, follow-up rate of patients, and patients’ appraisal. After dealing with problems of endogeneity and missing variables through the PSM method, independent variable replacement, and alternation of regression model, the conclusion is still robust. Besides, it was found that as an important external resource, patients’ support of A.I. applications influences the mechanism between A.I. and residents’ job performance. Finally, the job performance of female residents empowered by A.I. is much better than that of their male counterparts under the same conditions. However, no heterogeneity has been identified between A.I.’s impact on the job performance of medical practitioners and clinical medical technicians.

Recently, the contradiction between the surge in workload and the relative lack of medical resources in healthcare has been increasingly aggravating. The application of A.I. tools, benefiting from the development of digital technology, can significantly alleviate this contradiction. It can help improve the job performance of healthcare providers, as validated by this study. Therefore, the A.I. application in the medical system should be vigorously promoted to the extent that conditions allow. But also pay attention to the scope and extent of applications. On April 20, 2022, the National Health Commission of the People’s Republic of China issued the ‘National Restricted Technology Catalog and Clinical Application Management Specifications (2022 version)’, which establishes the list of medical technologies restricted by hospitals at all levels, including A.I. tools. This is significant positive news for the popularisation of A.I. tools in China, including the healthcare field. We hope, under this direction, more and more practical frameworks, policies and regulations related to the A.I. applications in all sectors (including the healthcare field) can be published in the future, which will pave the way for A.I. popularisation. From the organisational level, hospitals should strengthen the A.I.-relevant training for healthcare providers, provide A.I. tools within their capability, and perform an excellent job in the publicity of A.I. applications for patients and healthcare providers, aiming to create a thorough hardware and software environment for providers to apply the A.I. better while working. From the individual level, based on the concept of lifelong learning, healthcare providers should try to optimise their knowledge structure and master new intelligent technology.

This study, however, has some limitations. First, the sample size is limited, but the validity of the study is ensured as the questionnaire is filled under the careful guidance of our research group; moreover, the results have passed all the robustness tests. Second, the study is undertaken within one region in China, which cannot reflect the situation in other regions or other countries. However, since China’s AI has developed rapidly in recent years, our findings may be equally applicable to developed countries and some developing countries with rapid AI development. Third, the research sample is limited to the healthcare providers, without consideration of other groups. Fourth, this study is based on cross-sectional data, which does not reflect dynamic changes in effects. Fifth, via the PSM approaches etc., we have verified the robustness of the results so that there is no discussion on sample size adequacy, power analysis and representativeness in this study. However, it is also a limitation that needs to be addressed. Sixth, the bias type, the mitigation strategies, and the implications of the bias on the findings have not discussed in detail. In future research, cross-regional and cross-national studies with panel data can be made; more groups with the potential for applying A.I. need to be considered; the bias type, the mitigation strategies, and the implications of the bias on the findings may need to be included. Moreover, given the considerably growing of AI, the in addition to exploring the impact of AI application, future research can also investigate the effects of human-computer interaction on doctors’ work outcomes and mental health.

## Data availability statement

The original contributions presented in the study are included in the article/supplementary material, further inquiries can be directed to the corresponding authors.

## Author contributions

QZ: Conceptualization, Formal analysis, Investigation, Methodology, Supervision, Writing – original draft, Writing – review & editing. YJ: Funding acquisition, Writing – review & editing. XX: Data curation, Software, Validation, Writing – original draft.
